# Sequential Fabrication of a Three-Layer Retina-like Structure

**DOI:** 10.3390/gels10050336

**Published:** 2024-05-15

**Authors:** Yahel Shechter, Roni Cohen, Michael Namestnikov, Assaf Shapira, Adiel Barak, Aya Barzelay, Tal Dvir

**Affiliations:** 1 Shmunis School of Biomedicine and Cancer Research, Faculty of Life Science, Tel Aviv University, Tel Aviv 6997801, Israel; yahelshe@gmail.com (Y.S.); cohenroni8@gmail.com (R.C.); michael.names@gmail.com (M.N.); 0528788@gmail.com (A.S.); 2 Department of Biomedical Engineering, Faculty of Engineering, Tel Aviv University, Tel Aviv 6997801, Israel; 3 Sagol School of Neuroscience, Tel Aviv University, Tel Aviv 6997801, Israel; 4 Division of Ophthalmology, Tel Aviv Medical Center, Tel Aviv 6423906, Israel; adielbarak@gmail.com; 5 Sackler Faculty of Medicine, Tel Aviv University, Tel Aviv 6997801, Israel; 6 The Center for Nanoscience and Nanotechnology, Tel Aviv University, Tel Aviv 6997801, Israel; 7 Sagol Center for Regenerative Biotechnology, Tel Aviv University, Tel Aviv 6997801, Israel

**Keywords:** age-related macular degeneration, tissue engineering, retina, 3D bio-printing

## Abstract

Tissue engineering is considered a promising approach to treating advanced degenerative maculopathies such as nonexudative age-related macular degeneration (AMD), the leading cause of blindness worldwide. The retina consists of several hierarchical tissue layers, each of which is supported by a layer underneath. Each of these layers has a different morphology and requires distinct conditions for proper assembly. In fact, a prerequisite step for the assembly of each of these layers is the organization of the layer underneath. Advanced retinal degeneration includes degeneration of the other retina layers, including the choroid, the retinal pigmented epithelium (RPE), and the photoreceptors. Here, we report a step-by-step fabrication process of a three-layer retina-like structure. The process included the 3D printing of a choroid-like structure in an extracellular matrix (ECM) hydrogel, followed by deposition of the RPE monolayer. After the formation of the blood vessel–RPE interface, the photoreceptor cells were deposited to interact with the RPE layer. At the end of the fabrication process, each layer was characterized for its morphology and expression of specific markers, and the integration of the three-layer retina was evaluated. We envision that such a retina-like structure may be able to attenuate the deterioration of a degenerated retina and improve engraftment and regeneration. This retinal implant may potentially be suitable for a spectrum of macular degenerative diseases for which there are currently no cures and may save millions from complete blindness.

## 1. Introduction

Age-related macular degeneration (AMD) is a chronic disease of the central retina (macula), and it is the leading cause of blindness in Western countries [1]. The late stages of AMD are characterized by either choroidal neo-vascularization or geographic atrophy (GA). GA is characterized as a sharply defined area in the macula in which there is atrophy of the choriocapillaris, retinal pigment epithelium, and photoreceptors. Currently, there is no available cure for GA [2]. Recently, the FDA approved two complement inhibitor drugs to treat GA; however, these therapies are only able to slow down the progression of the disease to some extent and are not curative [3]. In a healthy retina, the layers are organized in a hierarchical pattern in which each layer is cardinal for the function and survival of the next [4]. The choriocapillaris are the closest to the Bruch’s membrane and the pigmented epithelial layer, and they supply oxygen and nutrients to the outer retina. Next, the retinal pigmented epithelial cells (RPE) provide the cardinal metabolic support to the photoreceptor cells on top. In AMD, as well as in other maculopathies, this symbiotic relationship and structure of the choriocapillaris/RPE/photoreceptors is lost [4,5]. 

Tissue engineering involves the design and creation of functional living tissues and organs from cells and biomaterials using engineering principles. Throughout the years, researchers have developed various fabrication technologies and approaches that have the potential to transform the field of medicine, including 3D printing [6], electrospinning [7], and molded scaffolds [8]. Aiming to restore vision loss due to RPE degradation, in recent years, tissue engineering approaches such as RPE and photoreceptor cell injection or the transplantation of pre-engineered retinal tissue parts have shown promising results [9,10,11]. In two cell injection studies, purified photoreceptors or RPE or progenitor cells [12,13] were injected into a wide area in the retina and could directly contact host cells [14,15]. However, in such cases, the cells could not form structured layers that may have assisted in maturation [14,15]. Contrary to this, the transplantation of RPE cell sheets has allowed for the delivery of a structured, mature layer of the RPE, which could better survive and properly interact with the host tissue [16]. For example, a groundbreaking clinical trial using autologous, iPSC-derived RPE for the treatment for dry age-related macular degeneration is currently ongoing [17]. However, since AMD is typically diagnosed at a late stage, when patients already suffer from distorted vision or central visual field defects due to photoreceptor loss [18], replacing the RPE layer alone can only support remaining photoreceptors and cannot restore lost vision. Furthermore, in advanced cases where atrophy includes degeneration of the choriocapillaris, it is essential to engineer a triple-layer tissue which includes the choriocapillaris, RPE, and photoreceptors. We note that the co-culture of RPE and photoreceptor cells is challenging, as these cells require different molecules for initial cell assembly. It is important to ensure that the different cell types in a co-culture system maintain their distinct identities and functions. This may involve using cell-specific culture media and supplements to support the growth and differentiation of each cell type. For example, endothelial cells require factors such as vascular endothelial growth factor (VEGF), basic fibroblast growth factor (bFGF), epidermal growth factor (EGF), and platelet-derived growth factor (PDGF). However, RPE cells also require insulin-like growth factor 1 (IGF-1), and photoreceptors require brain-derived growth factor and ciliary neurotrophic factor for their growth [19].

Research carried out by our group in the past utilized a personalized ECM-based hydrogel as a bio-ink for advanced 3D printing techniques [20,21]. The combination of the hydrogel and a patient’s own cells was used to print thick, vascularized, and perfusable patches that fully matched the immunological, biochemical, and anatomical properties of the patient. Moreover, the technology was used for the 3D printing of volumetric structures, such as a small-scale human heart [20]. This research utilized a decellularized ECM hydrogel derived from omentum tissue, which is a highly vascularized peritoneum with great regenerative properties. Furthermore, the omentum can be easily and safely removed from the patient in a minimally invasive procedure from. Unlike commercially available collagen-based hydrogels (such as GelMA), human ECM-based hydrogels consist of several molecules that are relevant to cell growth and tissue assembly, and most importantly, they match the immunological and biochemical properties of the patient [22].

Three-dimensional (3D) printing is an all-inclusive term for a variety of methods that use digital data to produce 3D objects made of various materials. The 3D bioprinting method is a highly regarded method in tissue engineering, as it enables effective control over the scaffold’s fabrication and cell distribution. Bioprinting can roughly be divided into the following three major categories: those based on light [23], inkjet [24], and extrusion [25] techniques.

Although it may be regarded as inferior in terms of resolution relative to inkjet and light-based bioprinting techniques, in this research, the extrusion method was chosen because it allows an optimal combination of precision and the ability to deposit viscous bio-inks containing high cell densities while maintaining excellent cell viability [26]. 

**Figure 1:**
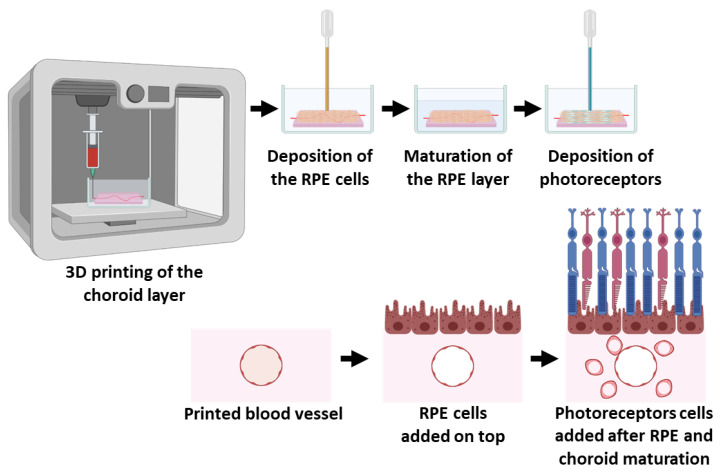
Schematics of the fabrication process. The blood vessel network was 3D-printed. After initial cell assembly, the RPE cells were deposited on top of the printed structure. Following 7 days of cultivation for the RPE monolayer assembly, photoreceptors were deposited to interact with the apical membrane of the RPE.

Here, we report a sequential fabrication of a three-layer retina-like structure (Figure 1). Initially, a blood vessel network was 3D-printed and cultivated for a short period of time for initial cell assembly. In the second stage, RPE cells within a thin layer of the ECM hydrogel were deposited onto the blood vessel network to form the choroid-RPE interface. After 7 days of cultivation, which allowed for the RPE cells’ assembly into a polarized monolayer, the photoreceptor cells were deposited to interact with the apical membrane of the RPE cells. Each layer was characterized separately for its morphology and expression of specific markers. Finally, the integration of the layers and the entire retina-like structure were investigated.

## 2. Results and Discussion

### 2.1. Fabrication of the ECM-Based Hydrogel

**Figure 2:**
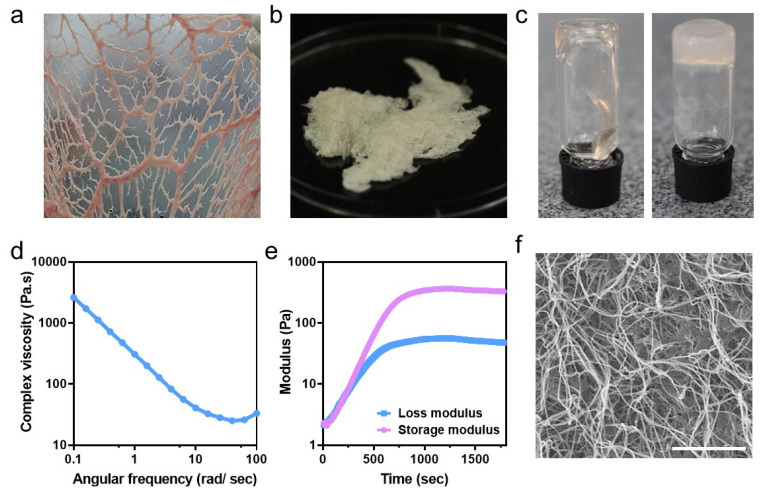
Fabrication of the ECM-based hydrogel. (**a**) Native porcine omentum. (**b**) Decellularized omentum. (**c**) ECM hydrogel. The ECM formed a weak gel at room temperature (left) and became a viscous, crosslinked hydrogel at 37 °C (right). (**d**,**e**) Rheological measurements of the 1% ECM hydrogel. (**d**) Post-gelation frequency sweep. (**e**) Time sweep at 37 °C. (**f**) SEM image of the hydrogel. Scale bar = 5 μm.

To fabricate the ECM hydrogel, we used an omental ECM. The omentum is a fatty tissue containing blood vessels and glycosaminoglycans and has remarkable regenerative capabilities [16]. This tissue can be taken from patients using a relatively easy, minimally invasive procedure to generate a personalized biomaterial [22]. As a proof of concept, omental tissues were obtained from porcine subjects and subjected to a decellularization procedure, preserving the ECM proteins (Figure 2a,b). The decellularized tissue was then processed into a thermoresponsive weak, liquid-like hydrogel at room temperature that became a viscous, physically crosslinked hydrogel under physiological conditions (Figure 2c–e). These properties enabled the hydrogel to be manipulated and structured prior to gelation. Additionally, SEM images revealed that the hydrogel was composed of ECM nanofibers that may encapsulate cells and promote multiple anchoring sites for cell adhesion (Figure 2f).

### 2.2. Engineering the Choroid Layer

**Figure 3:**
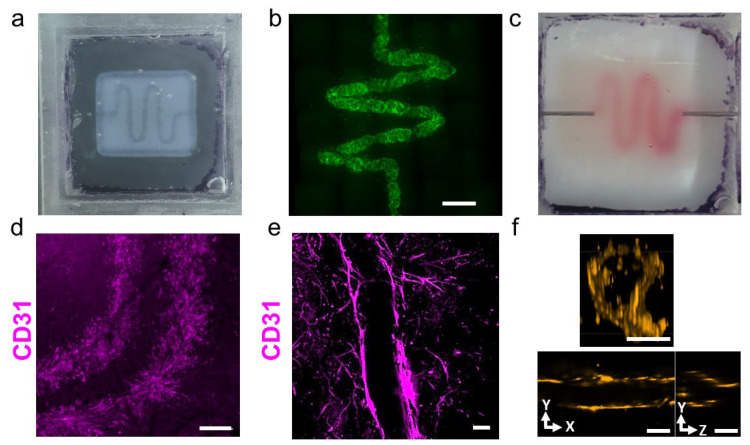
Engineering the choroid layer. (**a**) The printed structure. (**b**) The GFP-HUVECs formed a vessel-like structure in the culture. (**c**) Printed vessel during perfusion. (**d**) Two blood vessels printed in parallel. Scale bar = 500 μm. (**e**) The endothelial cells migrated out of the lumen to form a capillary bed. Scale bar = 100 μm. (**f**) Imaging of RFP-HUVECs printed in a vessel-like structure to form an open lumen (day 14). Scale bar = 100 μm.

Recently, we have shown that when mixed with cells, this thermoresponsive hydrogel can serve as a bioink for printing vascularized tissues [20]. Here, we sought to exploit this hydrogel for printing the choroid, which is the blood vessel layer supporting the outer retina. To generate a blood vessel with a 300 µm diameter and a capillary bed, endothelial cells were printed at room temperature in a sacrificial material based on particulate alginate and xanthan gum [27] which was surrounded by a printed ECM hydrogel (Figure 3a). During the gelation of the ECM hydrogel, the sacrificial material supported it, retaining its structure. As the sacrificial material properties did not provide cell anchors, the cells adhered to the surrounding ECM hydrogel. After complete gelation at 37 ˚C, the sacrificial material was enzymatically digested and removed, leaving the endothelial cells in a vessel-like structure with an internal diameter of approximately 300 μm (Figure 3b). To ensure sufficient nutrients and oxygen transfer from the printed structure, the blood vessel network was connected to a peristaltic pump, and proper perfusion was demonstrated (Figure 3c). The combination of convection through the vessel by perfusion and diffusion from the vessels into the hydrogel ensured a proper mass transfer throughout the fabricated structure [20]. Additionally, to facilitate the formation of a capillary bed, two blood vessels were printed in parallel (Figure 3d) to promote endothelial cell migration in the ECM hydrogel (Figure 3e). Finally, to ensure that the endothelial cells indeed lined up with the printed lumen, confocal microscope imaging was performed (Figure 3f).

### 2.3. Engineering the RPE Layer on Top of the Printed Vascular Layer

**Figure 4:**
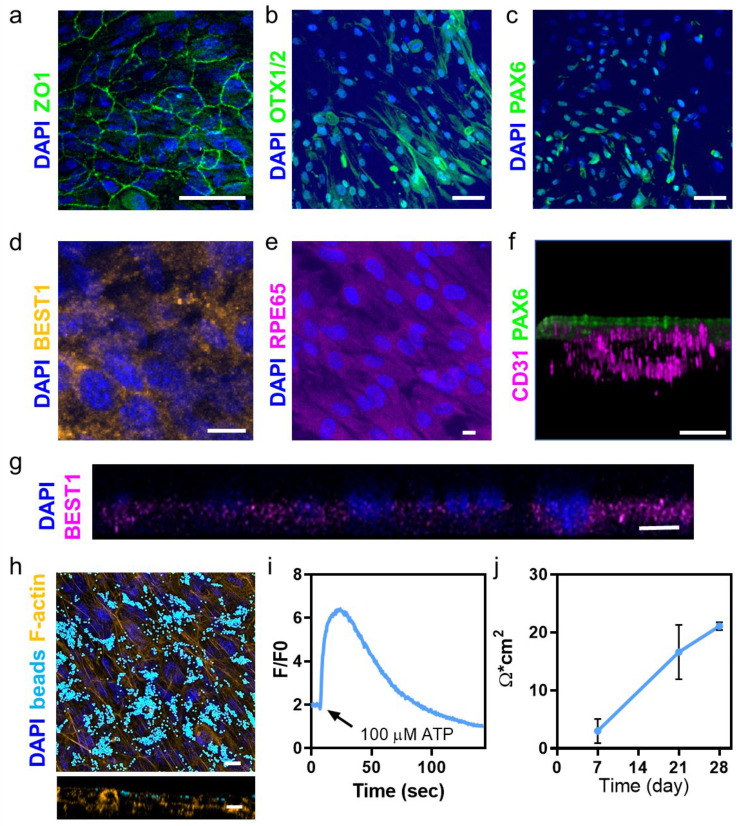
Engineering the RPE layer on top of the printed vascular layer. (**a**) The RPE cells in co-culture with endothelial cells expressed ZO1 (green). F-actin appears in orange and nuclei in blue. Scale bar = 50 μm. (**b**–**e**) RPE-specific markers. (**b**) OTX1/2 (green). Scale bar = 50 μm. (**c**) PAX6 (green). Scale bar = 50 μm. (**d**) BEST1 (pink). Scale bar = 10 μm. (**e**) RPE65 (pink). Scale bar = 50 μm. (**f**) A cross-section view of the blood vessel–RPE layer construct (day 28). Scale bar = 150 μm. (**g**) Polarity of the cells was demonstrated by the location of BEST1 (pink) within the RPE layer. Scale bar = 5 μm. (**h**) Phagocytosis of the latex fluorescent beads by the RPE cells after 20 days of culture of the blood vessel–RPE layer construct. Scale bar = 10 μm. (**i**) Calcium change in response to the ATP addition to the blood vessel–RPE construct (presented as a change from the baseline fluorescence; F/F0). (**j**) TEER measurements across the RPE layer.

Proper interaction between the RPE cells and photoreceptors requires initial maturation of the RPE in a polarized monolayer [28]. Therefore, a step-by-step process was needed, where the RPE layer was first assembled and matured before continuing to engineer the photoreceptor layer. The RPE layer was deposited dropwise on top of the printed choroid and allowed to self-organize and mature for 7 days. We then assessed the morphology of the RPE monolayer by analyzing the formation of tight junctions between the cells. Tight junctions play an important role in the barrier function of RPE cells, helping to maintain a proper balance of nutrients and waste products within the retina [29]. As shown, immunostaining for the tight junction marker ZO1 confirmed RPE monolayer junctional maturity (Figure 4a). Moreover, co-culturing the cells with the printed blood vessels promoted features characteristic of RPE cells, including defined cell borders, an overall ‘cobblestone’ appearance [30], and the expression of key RPE markers, such as OTX1/2, PAX6, BEST1, and RPE65 [31] (Figure 4b–e). The interface between the printed blood vessels and the RPE layer was demonstrated by co-staining the construct for CD31 and PAX6 (Figure 4f). The basolateral localization of BEST1 demonstrated the polarity of the RPE monolayer (Figure 4g), which was essential for nutrient transport and waste disposal between the photoreceptors and the choroid. If the polarity of the RPE cells was disrupted, it would lead to a misfunctioning retina [32]. 

Normally, in a healthy, functioning retina, phototoxic damage affects the outer segment of photoreceptors and the cells undergo a continuous turnover, where the underlying RPE monolayer phagocytes the tips of the outer segment [33]. Therefore, to assess the function of the engineered RPE layer, we next incubated the cells with fluorescent latex beads (d = 1 µm), and the ability of the engineered RPE layer to perform phagocytosis was demonstrated (Figure 4h). Moreover, several key RPE functions, such as the transport of nutrients and waste products across it, the maintenance of the blood–retinal barrier, and the regulation of cell growth and differentiation, are regulated by calcium ions [34]. Therefore, we next sought to assess calcium signaling. Calcium signaling in RPE cells is activated by light-induced increases in adenosine triphosphate (ATP) in the subretinal space, affecting, for instance, the regulation of the hydration and chemical composition of the subretinal space and the adhesion of the retina [35]. As shown, the engineered RPE layer reacted to ATP with the activation of the calcium-signaling pathways (Figure 4i). The addition of ATP immediately increased intracellular calcium levels in a significant manner, followed by a slow decrease to the baseline. Finally, to demonstrate that the RPE layer formed a barrier when cultured on top of the ECM hydrogel containing the blood vessels, trans-epithelial electrical resistance (TEER) measurements were performed during the 4 weeks of cultivation. A TEER value is a physiological index used to evaluate the permeability of a paracellular pathway by measuring the transport rate of ions or macromolecules. As shown, the TEER value significantly increased after 3 weeks of culture and was maintained until 4 weeks at 21.09 ± 0.67 Ω × cm^2^ (Figure 4j). A similar TEER value was measured for this cell line on different coatings [36].

### 2.4. Fabrication of the Three-Layer Retina-Like Structure

**Figure 5:**
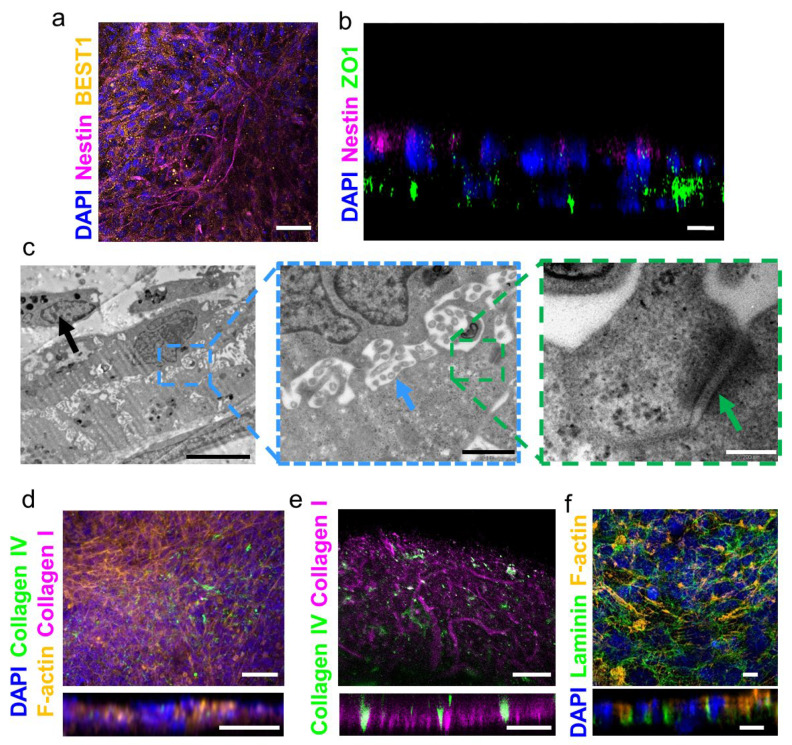
Fabrication of the three-layer retina-like structure. (**a**) Taken on day 27, an image of RPE cells expressing BEST1 (yellow) in the co-culture with endothelial cells and photoreceptors expressing nestin (pink). Scale bar = 50 μm. (**b**) A cross-section view of the RPE–photoreceptors construct, where the RPE expressed ZO1 (green) underneath photoreceptors cells that expressed nestin (pink). Scale bar = 10 μm. (**c**) TEM images of ARPE-19 cells after 104 days in the tri-culture of HUVECs and 661 w cells displaying pigmentation (black arrow), microvilli (blue arrow), and desmosomes (green arrow). Left image scale bar = 5 μm, middle image scale bar = 1 μm, and right image scale bar = 200 nm. (**d**–**f**) Immunostaining for the Bruch’s membrane proteins. (**d**) Collagen I (pink) and collagen IV (green). The cells appear in yellow (F-actin) and the nuclei appear in blue. Scale bar = 50 μm. (**e**) Non-cellular control of collagen I and IV. (**f**) Laminin staining. The cells appear in yellow (F-actin) and the nuclei appear in blue. Scale bar = 10 μm.

After 7 days, when the RPE cells had already formed a mature monolayer and a close interface with the blood vessel network underneath, photoreceptor cells (661 w cells) were seeded on top of the construct and cultivated for up to 94 days. Immunostaining confirmed the maturity of the RPE layer that expressed BEST1 and the presence of nestin-expressing photoreceptors (Figure 5a). Furthermore, a cross-section image of the construct revealed the assembly of the photoreceptors on top of the RPE layer (Figure 5b). Such localization of the cells is crucial for the viability and maintenance of the photoreceptors as they are supported by the RPE layer. Furthermore, cultivation of the three-layer retina-like structure for more than 100 days revealed key ultrastructural features of RPE cells (Figure 5c), including pigmentation (black arrow), microvilli (blue arrow), and desmosomes (green arrow), which are important for RPE cell function [37]. For example, melanosomes are pigment particles within RPE cells that give them their characteristic dark color. These pigment granules help to absorb light, reducing any stray light that may interfere with vision within the retina. Microvilli are small, finger-like protrusions on the surface of RPE cells that help to increase the surface area available for the exchange of nutrients, waste products, and signaling molecules between the RPE and photoreceptors. Desmosomes are special types of cell–cell junctions that help to anchor RPE cells to each other and to photoreceptors. By providing structural support and stability to the RPE layer, these junctions help to prevent damage to photoreceptors during eye movements. Additionally, desmosomes regulate the exchange of materials between RPE cells and photoreceptors, helping to maintain their health and function [38,39]. The existence of these features within the supporting RPE layer may provide further evidence for its maturation [32,33].

Bruch’s membrane is a thin, extracellular matrix layer that separates the retina from the choroid in the eye. It is composed of collagen I and IV, laminin, and other proteins, and it is an important structural element in the eye. The existence of this structure helps to maintain the shape of the retina, and it provides a substrate for the attachment of the RPE cells [40]. Therefore, we next sought to examine the existence of this ECM layer within the three-layer structure and its interaction with the cells. Immunostaining for ECM proteins of the Bruch’s membrane confirmed their existence between the RPE and the printed choroid (Figure 5d-f). As shown, the barrier between the RPE and blood vessel layers contained collagen I and IV (Figure 5d-e). Another key component of the Bruch’s membrane is laminin, which crosslinks the collagen fibers, structuring them as a 2D mesh [41]. As shown, the formed Bruch’s membrane contained laminin, expressed in the basolateral side of the RPE monolayer (Figure 5f). Overall, the detection of these ECM proteins indicated that our 3D model enabled the creation of Bruch’s membrane-like structures in vitro, and this enabled close interactions between the RPE cells and choriocapillaris. Such a structure can replace a damaged Bruch’s membrane in a patient, as well as support the cells of the retina and provide them with a proper environment for survival and function.

## 3. Conclusions

To summarize, when the complex choriocapillaris/RPE/photoreceptors malfunction, severe retinal degeneration can ultimately result in irreversible vision loss. Currently, treatment methods only address exudative AMD, leaving 90% of AMD patients with nonexudative and similar maculopathies with central atrophy with no curative treatment. As biofabrication technologies have advanced, complex cellular and tissue structures with high physiological relevance have been able to be engineered. Here, we aimed to develop a process for fabricating a three-layer retina-like structure. As a proof of concept, the fabricated structure was composed of human endothelial cells, RPE cells, and photoreceptor cell lines and an ECM-based hydrogel. As the three cell types require different cultivation conditions for their initial assembly and maturation, step-by-step tissue fabrication was performed. Initially, a blood vessel network was 3D-printed. Following this, RPE cells were deposited on top of the vasculature layer. The cells were allowed to assemble into a polarized monolayer, and only then, the photoreceptor cells were added to interact with the apical membrane of the RPE layer. The entire structure was further cultured for maturation. In the future, such retina-like structures may be able to attenuate the deterioration of a damaged retina and, in theory, due to the existence of the photoreceptors, regenerate it. Future experiments should focus on using pluripotent stem-cell-derived RPE cells and photoreceptors to prepare a personalized autologous retinal implant in order to reduce anti-graft immune responses. Furthermore, the efficacy of such fabricated retina-like structures should be assessed in an animal model of macular degeneration.

## 4. Materials and Methods

### 4.1. Bio-Ink Preparation

#### 4.1.1. Extracellular Matrix Hydrogel Production

As described previously [20], the omentum ECM hydrogel was prepared and kept at 4 °C in the liquid stage until printing. An omental tissue (Kibbutz Lahav, Israel) was agitated in a hypotonic buffer (0.2 M Tris-HCl, 0.5 M EDTA, and 1 M phenylmethanesulfonyl-fluoride) at pH 8.0 for 1 h. The tissue was then subjected to three cycles of freeze-thaw (−80–37 °C) using the same buffer. To dehydrate the tissue after the last cycle, it was washed once in 70% ethanol for 30 min, then it was washed three times in 100% ethanol for 30 min. Polar lipids were extracted from the tissue via three 30 min washes with 100% acetone. The a-polar lipids were extracted using a 60:40 hexane:acetone solution in three 8 h incubations. After defatting, the tissue was gradually rehydrated and degraded with 0.25% Trypsin-EDTA (solution B, Biological Industries, Beit Haemek, Israel) overnight at room temperature. The tissue was washed with PBS before incubation with 1.5 M NaCl for 24 h, which included three solution changes. After 24 h, the tissue was washed in a solution containing 50 mM Tris (pH 8.0) and 1% Triton X-100 (Sigma-Aldrich, Rheovot, Israel) for 1 h. The decellularized tissue was washed in PBS, followed by DDW, and then frozen (−20 °C) and lyophilized. After lyophilization, the decellularized omentum was ground into a coarse powder using a Wiley Mini–Mill and frozen until further use.

The decellularized omentum ECM (dECM) powder was enzymatically digested using 1 mg/mL of porcine pepsin (Sigma, 3200–4500 units mg^−1^ protein) in 0.1 M HCl prior to printing. The final concentration of dECM was 1% (*w*/*v*). The dECM was digested for 64–72 h at RT under constant stirring until the liquid was homogenous, with no visible particles. The pH was adjusted to 7.2–7.4 using 5 M NaOH to terminate pepsin activity, and the salt concentration was adjusted to physiological levels using DMEM-F12.

#### 4.1.2. Sacrificial Material Preparation

The sacrificial material was generated as per the method described earlier [27]. An aqueous solution was prepared that contained 0.32% (*w*/*v*) sodium alginate (PROTANAL LF 200 FTS, kindly provided by FMC BioPolymer), 0.25% (*w*/*v*) pre-treated XG, 37.5 mM sodium chloride, and 9.56 mM calcium carbonate (in suspension, Sigma-Aldrich). While constantly stirring, the mixture was supplemented with freshly prepared D-(+)-gluconic acid δ-lactone (GDL, Sigma-Aldrich, Rheovot, Israel) that had been pre-dissolved to achieve a final concentration of 19.15 mM. This led to a gradual decrease in pH, which caused the calcium carbonate to gradually dissolve, releasing the calcium ions that crosslink the alginate. After increasing the viscosity of the solution to a level that prevented calcium carbonate precipitation, the mixture was incubated at room temperature for 24 h without stirring. DDW was added at 4 times the volume of the resulted hydrogel, followed by homogenization at 25,000 RPM for 2 min (HOG-020 homogenizer with GEN-2000 generator probe, MRC ltd, Israel). The homogenate was incubated at 4 °C for 24 h, and then it was centrifuged at 15800 g for 20 min. The pellet was washed in DDW by resuspending it through vigorous vortexing. It was then recentrifuged and resuspended in Dulbecco’s modified Eagle medium (DMEM)/F12 (HAM) 1:1 culture media (Biological Industries, Beit Haemek, Israel) before undergoing another round of centrifugation, following which the supernatant was discarded. Next, 1.1% (*w*/*v*) XG in DDW was added to the pellet at a 1:20 volume ratio to reach a final concentration of ~0.05% (*w*/*v*), followed by vigorous vortexing to homogenize the mixture. The mixture was incubated at RT for 3–4 days or at 4 °C for 6–8 days. After this period, the mixture could be immediately used for printing or stored at room temperature or 4 °C for later use.

### 4.2. Sacrificial Material Extraction

In order to extract the sacrificial material after printing, the printed disk was incubated at 37 °C for 30 min to cure the bioink. As a next step, alginate lyase was added to the growth medium (Sigma-Aldrich, 1 U ml^−1^) and the disk was incubated at 37 °C until the digested sacrificial material turned liquid.

### 4.3. Rheological Properties

Rheological measurements were conducted using a Discovery HR-3 hybrid rheometer (TA Instruments, New Castle, DE, USA) with a parallel plate geometry of 20 mm in diameter and a Peltier plate to maintain the sample’s temperature. The samples were loaded at a temperature of 4 °C and then heated to 37 °C to allow gelation. During the process, the oscillatory moduli of the samples were monitored at a fixed frequency of 1 Hz and a strain of 1%.

### 4.4. Cell Culture

The ARPE-19 cells were cultured in a Dulbecco’s modified Eagle medium (DMEM): F-12 (Ham’s) nutrient mixture (1:1), with sodium bicarbonate 1.2 g/L, Hepes 15 mM, and sodium pyruvate 55 mg/L, supplemented with 10% FBS, 1% L-glutamine, and a 1% penicillin-streptomycin solution (all from Sartorius). The cell medium was replaced twice per week. Primary human umbilical vein endothelial cells (HUVECs) (Lonza) were maintained in EGM2 (Lonza) and supplemented according to the manufacturer instructions. Immortalized GFP-labeled HUVECs were cultured in DMEM and supplemented with 10% FBS, 1% L-glutamine, and 1% penicillin-streptomycin, and the cell medium was replaced every other day. An early passage (12) of a 661 W photoreceptors cell line was a kind gift provided by Prof. Muyyad R. Al-Ubaidi, and the cells were grown in DMEM supplemented with 10% FBS, 1% L-glutamine, and 1% penicillin and streptomycin at 37 °C in 5% CO_2_. All cells were passaged using 0.05% trypsin (Sartorius). The printed structure with the ARPE-19, HUVECs, and 661 w was cultured in EGM2, and the media was replaced every other day. 

### 4.5. Perfusion Culture

A perfusion chamber was designed using an open-source design [42] and printed with a MAX X43 DLP printer (ASIGA). The implant was printed directly in the perfusion chamber and connected to a multichannel peristaltic pump (Ismatec, ISM93D) with a 27 G needle and Ismatec pump tubing (2-Stop, PharMed^®^ BPT, 0574-95723-12). The media was perfused at a rate of 104 µL/h.

### 4.6. Immunofluorescence Staining

The samples were washed twice with cold PBS after removing the culture medium. After that, the cells were fixed using 4% PFA in PBS for 20 min at room temperature and then washed three times with PBS. To permeabilize the samples, 0.1% Triton-X100 in PBS was added for 5 min, followed by three more washes with PBS. Next, the samples were treated with a blocking solution (2% BSA in PBS) for 1 h at room temperature. Primary antibodies were then diluted in a blocking solution according to the specifications shown in Table 1, and then they were added to the samples for 1 h at room temperature or at 4 °C overnight. The following secondary antibodies were incubated for 1.5 h: goat anti-rabbit Alexa 488 (Abcam, ab150077, 1:250), goat anti-mouse Alexa 647 (Abcam, ab150119, 1:250), and goat anti-mouse Alexa 555 (Abcam, ab150118). Phalloidin staining was performed by adding phalloidin conjugated to iFluor 647 (Abcam, ab176759, 1:1000) or phalloidin conjugated to iFluor 555 (Abcam, ab176756, 1:1000). For nuclei detection, the samples were incubated for 5 min with Hoechst 33258 (1:20; Sigma). Images were taken using confocal microscopy (Nikon Eclipse Ni, Tokyo, Japan) or an inverted fluorescence microscope (Nikon Eclipse TI).

### 4.7. Phagocytosis Assay

The ARPE-19 or hRPE cells were grown until confluence on dECM-coated 13 mm diameter glass cover slides in a 24-well plate, and the 1 μm carboxylate-modified polystyrene fluorescent yellow-green beads (Sigma) were prepared by washing them 3 times with sterile PBS to remove the sodium azide. The cells were incubated for 16 h at 37 °C in medium containing different concentrations of the beads. The samples were washed 3 times with PBS, fixated for 20 min with 4% PFA, and stained with Alexa-Fluor-647-conjugated phalloidin (Sigma). For the visualization of F-actin, the fluorescence signal was observed with a laser-scanning confocal microscope, and the phagocytotic cells were counted manually. Statistical significance was determined by an unpaired *t*-test.

### 4.8. Calcium Imaging

The Fluo-4-AM was prepared by adding 45 µL DMSO and 45 µL Pluronic-DMSO to a 50 µg vial of Fluo-4-AM. The Fluo-4-AM was diluted with HBSS at a ratio of 1:50, and the cells were incubated for 1 h at 37 °C. The cells were then washed for 30 min in HBSS at 37 °C. The sample was placed in an inverted microscope on a heating plate adjusted to 37 °C. Fluo-4-AM emission is at 506 nm and excitation is at 494 nM. Images were acquired at a rate of 3.15 Hz. Ten seconds after starting the recording, 100 µM ATP was added to the medium. The data were analyzed using ImageJ (NIH, version 1.5.3).

### 4.9. TEM

The TEM samples were fixated in 2.5% glutaraldehyde in PBS overnight at 4 °C. After multiple washes in PBS, the samples were post-fixed in 1% OsO4 in PBS for 2 h at 4 °C. The process of dehydration was performed using graded ethanol, which was then followed by embedding the sample in glycid ether. Thin sections were mounted on Formvar/carbon-coated grids, stained with uranyl acetate and lead citrate, and examined with a Jeol 1400-Plus transmission electron microscope (Jeol, Japan). The images were taken with SIS Megaview III and iTEM, which are the TEM imaging platforms used by Olympus.

### 4.10. SEM

Samples for SEM were fixated in 2.5% glutaraldehyde for 24 h at 40 °C, followed by a series of graded incubations in ethanol–water solutions ranging from 50–100% (*v*/*v*). The samples were dried using a critical point dryer, sputter-coated with gold using a Polaron E 5100 coating apparatus (Quorum Technologies, Lewis, UK), and then observed under a JSM-840A SEM (JEOL, Tokyo, Japan).

### 4.11. Trans-Epithelial Electrical Resistance (TEER)

The barrier properties of the ARPE-19 cells cultured on the dECM hydrogel were evaluated with TEER measurements. The ARPE-19 cells were seeded on 24-well inserts (Greiner) coated with the dECM hydrogel at a density of 1.7 × 10^5^ cells/cm^2^ and cultured for 4 weeks. The TEER was measured with a Millicell ERS-2 Voltohmmeter (Merck Millipore). TEER values (Ω × cm^2^) were calculated by subtracting the TEER value of a similarly coated well insert without cells and multiplying the result by the insert surface area. TEER values were obtained for 3 parallel samples and 3 technical replicates.

## Figures and Tables

**Table 1:** The primary antibodies used for the immunofluorescence staining.

Name	Working Dilution	Catalog Number	Manufacturer
Rabbit anti-PAX6	1:150	ab195045	Abcam
Mouse anti-BEST1	1:150	ab2182	Abcam
Rabbit anti-OTX1/2	1:150	ab21990	Abcam
Mouse anti-RPE65	1:100	NB100-355	Novus
Rabbit anti-ZO1	1:200	CST-13663S	Cell signaling
Mouse anti-CD31	1:250	P8590	Sigma
Chicken anti-nestin	1:2000	ab134017	Abcam
Rabbit anti-collagen IV	1:500	ab6586	Abcam
Rabbit anti-laminin	1:50	ab11575	Abcam
Mouse anti-collagen I	1:4000	MA126771	Invitrogen

## Data Availability

All data and materials are presented in this manuscript.
